# Probiotics in inflammatory bowel diseases: emphasis on mechanisms and clinical application

**DOI:** 10.3389/fmed.2025.1620079

**Published:** 2025-08-01

**Authors:** Junxiang Zhang, Xiaoman Zhang, Xiaoxin Cheng, Shijin Wang, Yangxuan Lv, Xu Zheng, Guangzhen Wu

**Affiliations:** ^1^Department of Urology, The First Affiliated Hospital of Dalian Medical University, Dalian, China; ^2^Department of Cell Biology, College of Basic Medical Science, Dalian Medical University, Dalian, China

**Keywords:** probiotics, inflammatory bowel disease, Crohn’s disease, ulcerative colitis, gut microbiota

## Abstract

Inflammatory bowel disease (IBD) is a group of diseases characterized by chronic intestinal inflammation including Crohn’s disease (CD) and ulcerative colitis (UC). In recent years, probiotics have attracted more and more attention as a potential adjuvant therapy. Probiotics can improve the symptoms and quality of life of IBD patients mainly by regulating intestinal microflora, regulating immune response, enhancing intestinal barrier function and exerting anti-inflammatory effect. However, although a large number of studies have explored the role of probiotics, there are still individual differences and uncertainties in clinical application. This paper reviews the mechanism, clinical effect and future prospect of probiotics in the treatment of IBD, and analyzes the existing clinical research and experimental data to provide reference for further research. Finally, this paper looks forward to the research direction of probiotics in the treatment of IBD, aiming at providing evidence for clinical practice.

## 1 Introduction

Inflammatory bowel disease (IBD) is a complex and challenging chronic inflammatory disease, which is characterized by repeated intestinal inflammation, including Crohn’s disease (CD), ulcerative colitis (UC) and unclassified inflammatory bowel disease (IBD-U) (1). These diseases will not only significantly affect the digestive function of patients, but also often accompany a series of disturbing symptoms, such as abdominal pain and diarrhea (2, 3), as well as weight loss, it may also lead to a series of parenteral manifestations, such as peripheral arthritis, oral ulcer, scleritis or erythema nodosum, which seriously affects the quality of life and daily activities of patients (4–6). The pathogenesis of IBD is extremely complicated, involving the interaction of many factors, including genetic factors (genes related to immune response and intestinal barrier integrity) (7–10), environmental factors (such as smoking, eating habits, drug use, social pressure and psychological factors) (11–16), and immune factors (including the functions of innate and adaptive immune pathways) (17–19). Epidemiological studies show that the prevalence of IBD is significantly different in different regions. The prevalence rate in North America and Europe is high and has stabilized in recent years, while the incidence rate in some East Asian countries is on the rise (20). At present, the treatment of IBD mainly includes drug therapy, nutritional intervention and surgical treatment. Commonly used drugs include aminosalicylic acid preparation, glucocorticoid, immunomodulator and biological preparation (21–24). However, these treatments are often accompanied by various side effects, and some patients do not respond well to these treatments, leading to the recurrence or aggravation of the disease (25–27). Therefore, it is very important to identify new treatment schemes for improving the prognosis of patients with IBD.

In recent years, with the in-depth study of intestinal microbiota, people have gradually found that microbiota plays an important role in the occurrence, development and rehabilitation of IBD. Probiotics, as a new treatment method, show broad application prospects (28, 29). Probiotics can help alleviate inflammation in many ways, such as regulating intestinal microflora, enhancing intestinal barrier function and regulating immune response, thus becoming an important supplement to the treatment of IBD (26, 30). Therefore, probiotics, as a new treatment strategy, may bring hope to patients with IBD, which deserves further research and clinical application exploration in order to improve the quality of life and overall health status of patients.

## 2 Probiotics: conceptual definition and systematic categorization

### 2.1 What are probiotics?

Probiotics are living microorganisms that can change the intestinal flora and bring health benefits to the host when ingested in sufficient quantities (31). The viability of probiotics confers a spectrum of health benefits to the host, chiefly manifested in their pivotal roles in sustaining gut homeostasis and promoting overall wellbeing. These effects are mediated through mechanisms that include modulation of the intestinal microbiota equilibrium, enhancement of host immune function, suppression of pathogen growth, and facilitation of efficient nutrient absorption (32–34).

In addition, the application of probiotics has been extended to many health fields, from the most basic improvement of intestinal health to enhancing the immune function of the body, showing positive research prospects (35–37). However, although the potential of probiotics in the health field has been widely recognized, misunderstandings among the public still need to be clarified. A common misunderstanding stem from oversimplified commercial propaganda that all probiotics are the same. In fact, the efficacy of probiotics has a high degree of strain specificity and host individual differences (38). Although probiotics and prebiotics have a good safety record, they still need to be cautious in specific health conditions or specific patient groups (39, 40).

### 2.2 Main types of probiotics and functions

The probiotic types listed in Table 1 are diverse and primarily encompass the following major categories: *Lactobacillus*, *Bifidobacterium*, and *Saccharomyces* (41).

**Table 1:** The main types and functions of probiotics.

	Strain	Function	References
Lactobacillus			
*L. rhamnosus* GG	LGG	Inhibit inflammatory responses, anti-cancer effects and promote skin wound healing.	(45–47)
*L. acidophilus*	CICC 6092, NCFM	Improve obesity and related diseases; its surface proteins can improve the tissue pathological damage caused by colitis.	(48, 49)
*L. casei*	LTL1361	Reducing the damage of pathogens to the intestines; its combination with DFC has anti-aging effects.	(50, 51)
Bifidobacterium			
*B. longum*	BB536	Improve chronic constipation in the elderly and inhibit obesity.	(52, 53)
*B. breve*	M-16v	Improve cognitive function, inhibit brain atrophy and suppress obesity.	(52, 54)
*B. bifidum*	ATCC29521	Affect intestinal inflammatory cells, downregulate the expression of GDNF, TLR-2, and TNF-α, and exert anti-inflammatory effects.	(55)
Saccharomyces			
*S. cerevisiae*	CNCM I-3856	Alleviate abdominal pain in IBS-C patients primarily suffering from constipation, and improve the quality of life for these patients.	(56)
*S. boulardii*	CNCM I-745	Quickly treat dysbiosis, reduce chloride secretion and treat diarrhea.	(57, 58)

*Lactobacillus*, as an important representative of probiotics (such as *Lactobacillus rhamnosus* GG (LGG), *Lactobacillus acidophilus* and *Lactobacillus casei*), have functional characteristics that exceed the traditional understanding of acid-producing and antibacterial effects. The latest research shows that *Lactobacillus* has multiple physiological functions, such as regulating the intestinal microecology, protecting the digestive system, and regulating the immune system (42–44). LGG can regulate immune responses, improve inflammatory responses, and prevent damage to colonic tissues (45). The extracellular vesicles released by *Lactobacillus* enhance the proliferation and migration abilities of epithelial and endothelial cells and promote the formation of endothelial tubes, showing therapeutic potential in skin wound healing (46). Besides, it can also improve cancer cell responses to anti-PD-1, thereby exerting anti-cancer effects (47). *L. acidophilus* can alleviate obesity and related diseases by improving endothelial dysfunction and gut microbiota imbalance through its anti-inflammatory properties (48). Chandhni et al. also indicated that the surface proteins in the *Lactobacillus acidophilus* NCFM strain can reverse the histopathological damage caused by colitis (49). The surface layer protein of *Lactobacillus casei* FB05 can reduce the harmful effects of *Escherichia coli* and *Salmonella* on the intestine by decreasing pathogen adhesion and inhibiting pathogen-induced apoptosis (50). Additionally, Ren et al. showed that *Lactobacillus casei* LTL1361 and dietary fiber complexes (DFC) alleviated age-related cognitive impairment and protected brain and gut functions. *L. casei* LTL1361 and DFC may serve as novel and promising human anti-aging agents (51).

*Bifidobacterium* (such as *Bifidobacterium longum*, *Bifidobacterium breve*, and *Bifidobacterium bifidum*) possesses beneficial homeostatic and anti-inflammatory immune regulatory characteristics. *Bifidobacterium longum* BB536 and *Bifidobacterium breve* MCC1274 may also reduce visceral fat and total body fat levels in healthy normal-weight and overweight adults, thereby helping to prevent obesity (52). In addition, Takeda et al. reported that *B. longum* BB536 can improve bowel movements and has a positive effect on chronic constipation in the elderly (53). *B. breve* MCC1274 can improve cognitive function and helps inhibit brain atrophy (54). *B. bifidum* exhibits anti-inflammatory effects. Yang et al. found that exogenous stimulation with lipopolysaccharide plus Interferon-γ (IFN-γ) induced increased expression of major histocompatibility complex class II (MHC-II) and CD86 in enteric glial cells (EGC), and further treatment with *B. bifidum* supernatant downregulated MHC-II expression. Additionally, *B. bifidum* downregulated the expression of glialcell-derived neurotrophic factor (GDNF), Toll-like receptor 2 (TLR-2), and tumor necrosis factor-α (TNF-α) in stimulated EGC (55).

*Saccharomyces*, such as *Saccharomyces cerevisiae* and *Saccharomyces boulardii*, are also important probiotics and should not be ignored. They not only help to maintain the balance of intestinal microflora, but also show the characteristics of resisting pathogenic microorganisms in some cases. Mourey et al. studied the effect of *Saccharomyces cerevisiae* CNCM I-3856 on constipation-predominant irritable bowel syndrome (IBS-C), and found that the response rate of patients receiving probiotic supplementation in relieving abdominal pain was significantly higher than that of placebo group (45.1% vs. 33.9%, *p* = 0.017). In addition, after 8 weeks of supplementary treatment, the overall quality of life score of the probiotic group was significantly higher than that of the placebo group (*p* = 0.047), indicating that probiotic I-3856 is helpful to improve abdominal pain and quality of life of IBS-C patients (56). Oral administration of *Saccharomyces boulardii* CNCM I-745 has no effect on the intestinal microflora of healthy subjects. However, under some conditions of intestinal flora imbalance, the application of *S. boulardii* CNCM I-745 can help to restore intestinal flora more quickly (57). In addition, *S. boulardii* CNCM I-745 can reduce chloride ion secretion, so it has a certain effect in treating diarrhea (58).

With the deepening of scientific research, the multiple functions of probiotics are gradually recognized. A large number of studies show that probiotics may have a positive effect on improving metabolic syndrome, diabetes, allergic diseases and intestinal infections (59–61). These results highlight the potential value of probiotics as an adjuvant therapy. These research results provide an important scientific basis for the application of probiotics in clinical treatment and show its great potential in modern medicine (Table 1).

### 2.3 Mechanisms of probiotics in IBD

**Figure 1:**
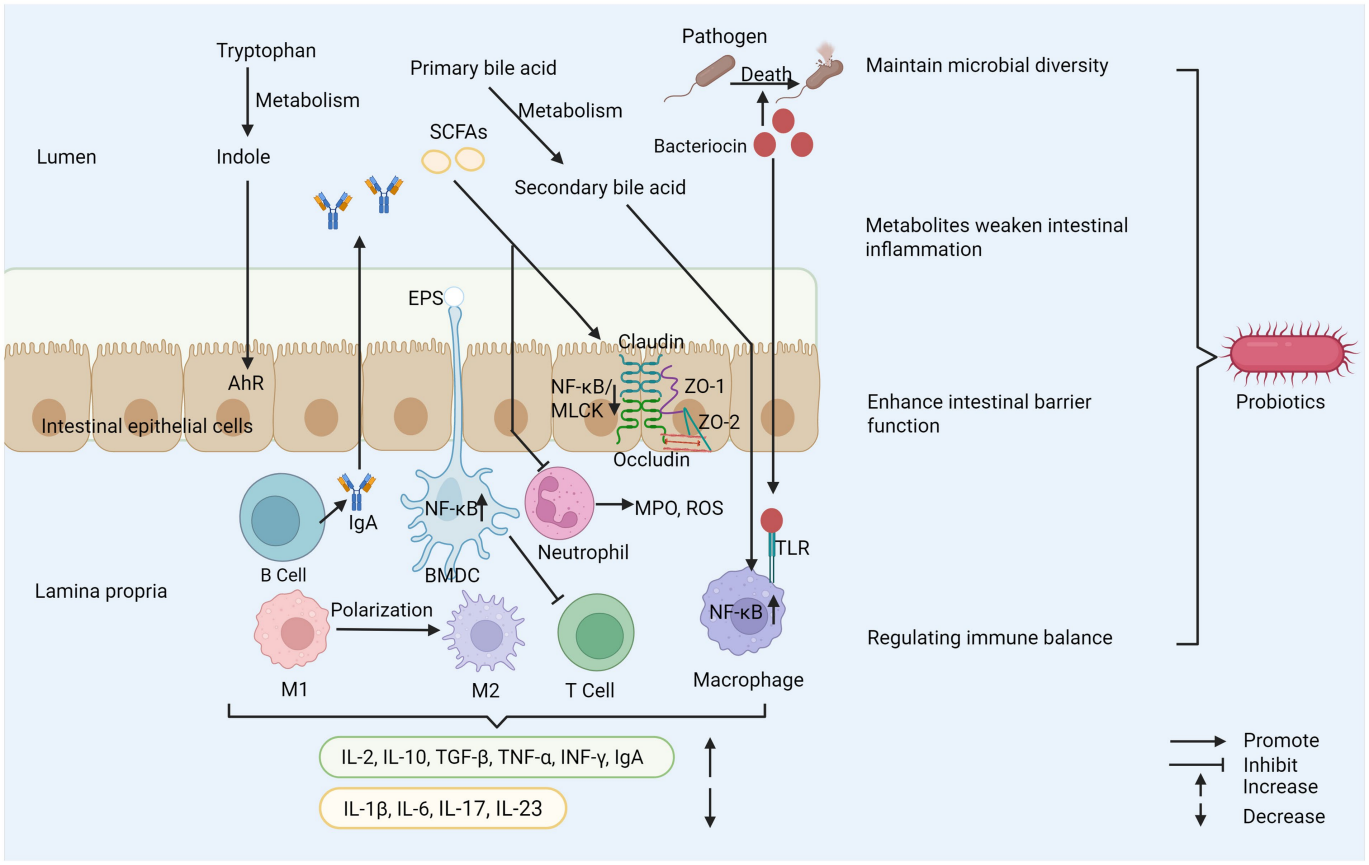
Probiotics play several important roles in maintaining health. (1) Probiotics inhibit the reproduction of pathogenic bacteria and maintain microbial diversity. (2) Probiotics regulate immune cell activity and cytokine secretion to help maintain immune balance. (3) Probiotics enhance the integrity of tight junctions between cells to strengthen intestinal barrier function. (4) Their metabolites can reduce intestinal inflammation. SCFAs, short–chain fatty acids; EPS, exopolysaccharide; AhR, aryl hydrocarbon receptor; ZO-1, zonula occludens-1; ZO-2, zonula occludens-2; MPO, myeloperoxidase; ROS, reactive oxygen species; TLR, toll-like receptor; M1, macrophage type 1; M2, macrophage type 2; BMDC, bone marrow-derived dendritic cell; MLCK, myosin light chain kinase.

The application potential of probiotics in the treatment of IBD has been paid more and more attention, which involves several key mechanisms. Its core mechanism involves many interrelated levels, far more than simple flora regulation. These key mechanisms together constitute the theoretical basis for probiotics to alleviate the pathological state of IBD, as shown in Figure 1 (62, 63).

#### 2.3.1 Regulation of gut microbiota

Healthy intestinal microflora not only has diversity, but also has good stability, and cooperates in many ways to jointly safeguard the health of the host (64). For example, intestinal microflora plays a vital role in food digestion and absorption, vitamin synthesis, immune system regulation and intestinal barrier protection (65, 66). A large number of studies show that probiotics can effectively restore the balance of intestinal microflora through various mechanisms, such as inhibiting the growth of pathogenic microorganisms, thus reducing the risk of infection (67). Probiotics can also enhance the diversity and stability of intestinal microflora by producing antibacterial substances and promoting the proliferation of beneficial bacteria (68). Microbial diversity is an important factor to maintain intestinal health. This diversity not only helps to improve digestive function, but also reduces the risk of intestinal inflammation (69, 70).

**Figure 2:**
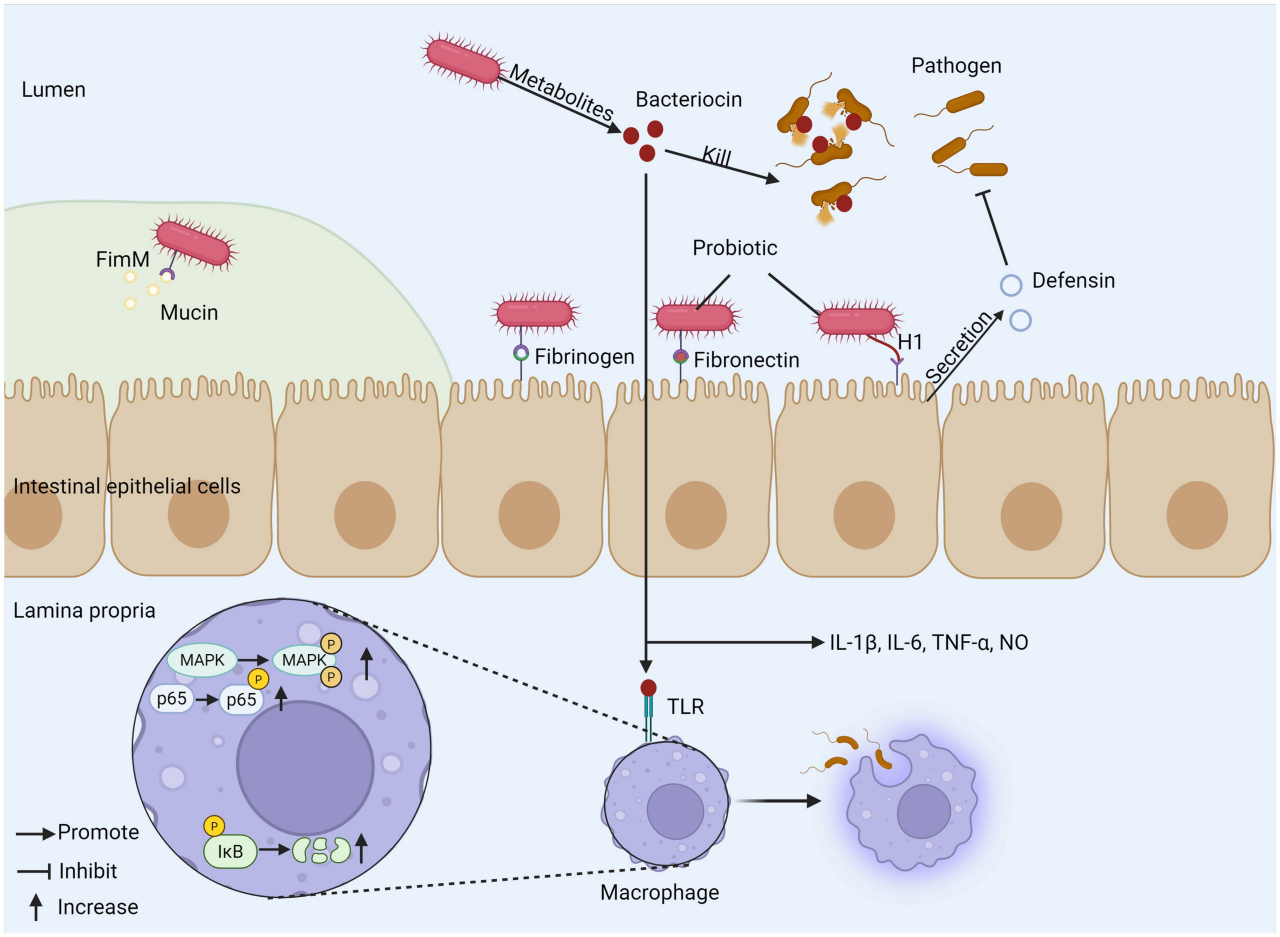
The mechanism of probiotics regulating gut microbiota. (1) Probiotics produce bacteriocins to kill pathogens and promote phagocytosis of macrophages, inducing cytokine release. (2) Probiotic FimM binds to mucin, fibronectin, and fibrinogen to inhibit pathogen invasion. (3) H1 flagellum compete with pathogens for binding sites and can stimulate epithelial cells to produce defensin. FimM, fimbrial m protein; MAPK, mitogen-activated protein kinase; TLR, toll-like receptor; I*κ*B, inhibitor of Kappa B.

For patients with IBD, the composition of intestinal microflora often changes significantly, which usually shows a decrease in microbial diversity and the number of some beneficial bacteria (71). For example, in the intestinal flora of patients with CD, the abundance of *Christensenellaceae*, *Coriobacteria* and *Faecalibacterium prausnitzii* decreased, while the abundance of *Actinobacteria, Veillonella* and *Enterobacteriaceae* increased. As for patients with UC, the abundance of *Eubacterium rectale* and *Akkermansia* decreased, while the abundance of *Enterobacteriaceae* increased (72). Some studies have found that patients with CD have a higher degree of flora imbalance than patients with UC (73). The root cause of IBD is not clear, but the imbalance of intestinal microflora, especially the decrease of abundance and diversity of specific genera, is considered as the inducement of IBD (74). Probiotics are widely regarded as a potential adjuvant therapy for IBD. They can effectively relieve inflammation by improving the composition of intestinal microflora, promoting the proliferation of beneficial bacteria and inhibiting the attachment of pathogenic microorganisms (75, 76). *Escherichia coli* Nissle 1917 (ECN-pE) can inhibit the growth of *Salmonella* and other pathogens. Its H1 flagella enables the probiotic to compete with pathogens for binding sites on host tissues effectively, thus inhibiting the adhesion and invasion of pathogens to intestinal epithelial cells. It can also directly stimulate intestinal epithelial cells to produce defensins and has anti-inflammatory effects. In addition, the gene encoding F1C fimbriae plays a key role in the continuous colonization of bacteria and its adhesion to intestinal epithelial cells (77). *Bifidobacterium longum* BBMN68 has a new surface adhesion protein called FimM (Fimbrial m protein), which is the main fimbrial protein subunit of *B. longum* BBMN68, which may be used as a surface adhesion monomer but cannot form a fimbrial-like structure, mucoprotein, fibronectin and fibrinogen are its adhesion receptors, mucoprotein is the main structural component of the mucus layer and provides a physical barrier to the intestinal epithelial surface, and normally FimM binds to mucin to prevent pathogens from entering the mucus layer. During pathogen invasion, FimM interacts with fibronectin and fibrinogen to inhibit pathogen adhesion (78), and FimM can produce various substances such as bacteriocin and biosurfactants for specific probiotic strains, which are harmful to pathogenic microorganisms (79, 80). Bacteriocins can also enhance immune function and inhibit the growth of pathogenic bacteria, and bacteriocin in *Bacillus subtilis* can induce interleukin-1β (IL-1β), IL-6, TNF-α, and nitric oxide release modulate innate immunity, enhance phagocytosis in mouse peritoneal macrophages, and induce phosphorylation of three mitogen-activated protein kinases (MAPK) (p38 MAPK, extracellular signal-regulated kinase 1/2 (ERK1/2), and c-Jun N-terminal kinase (JNK)) in macrophages. Aberrant activation of nuclear factor kappa B (NF-κB) signaling pathway plays a key role in the molecular pathological mechanism of IBD, and studies have shown that sublancin-stimulated nuclear factor kappa B (NF-κB) p65 and κB inhibitor (IκB-α) can enhance the phosphorylation of NF-κB p65 and degrade IκB-α (81). The two most important bacterial phylum in gastrointestinal tract, *Firmicutes* and *Bacteroides*, showed that the ratio of *Firmicutes/Bacteroides* (F/B) related to IBD decreased. *Firmicutes* have anti-inflammatory effect, which can slow down the progress of IBD, while *Bacteroides* can produce endotoxin, which shows pro-inflammatory characteristics and affects the production of cytokines, thus leading to IBD. Some bacteria from *Lactobacillus* and *Bifidobacterium*, such as *Lactobacillus reuteri* DSM 17938, *Lactobacillus plantarum* AN1 and fermented milk containing *Bifidobacterium*, can affect the F/B ratio, thus relieving IBD (82–84). It is worth noting that the form of probiotic preparation also affects its curative effect. Compared with a single strain, multi-strain probiotic preparation can regulate the intestinal flora more effectively, generally regulate the intestinal flora structure more comprehensively, restore its diversity and balance, and may produce superposition or synergistic effect through multiple mechanisms, thus further enhancing the therapeutic effect on IBD (85). Therefore, probiotics show great potential and value in the management of IBD by precisely regulating key inflammatory pathways such as NF-κB, reshaping disordered intestinal flora (especially correcting the imbalance of F/B ratio) and strengthening the intestinal barrier (Figure 2).

#### 2.3.2 Immune regulation

Probiotics exert their protective effects by modulating host immune responses, primarily reflected in promoting T cell differentiation, regulating cytokine levels, and increasing immunoglobulin A (IgA) levels (86–88). Probiotics may promote the interaction between co-stimulatory molecules CD80/86 on dendritic cells (DCs) and cytotoxic t-lymphocyte-associated protein 4 (CTLA-4), thereby leading to a weakened T cell response (89). Experimental evidence has shown that the administration of probiotics enhances the production of interleukin (IL)-10 and increases the proportion of regulatory CD4^+^ T cells expressing surface transforming growth factor-β (TGF-β) in the form of latency-associated peptide (LAP), also known as LAP^+^ regulatory T cells (Treg), whose presence depends on IL-10 production (90). Probiotics also increase the number of CD4^+^CD25^+^Foxp3^+^Treg cells, which can secrete inhibitory factors such as IL-2, IL-10, and TGF-β to regulate the balance of Th1/Th2 cells and maintain intestinal mucosal immunity (91). Additionally, it has been shown that LGG and *B. breve* may reduce the activity of IL-17 and IL-23, both of which play important roles in the formation and activation of Th17 cells. Different strains of *Lactobacillus* and *Bifidobacterium* produce TNF-α and IFN-*γ*, which can inhibit the proliferation of Th17 inflammatory cells (92).

Probiotics have the capacity to influence macrophage polarization, facilitating the transition from M1 to M2 macrophages, thereby contributing to the amelioration of colitis (93, 94), Furthermore, elements of the probiotic cell wall are implicated in the immunomodulation of DCs. Specifically, the interaction of capsular polysaccharides with TLR2 receptors on DCs induces T helper cells to secrete IL-10, consequently mitigating inflammation linked to colitis (94).

Probiotics modulate a variety of immune signaling pathways, such as JAK/STAT and NF-κB, thereby reducing the production of inflammatory cytokines (95, 96). For example, *Bifidobacterium* has been shown *in vitro* experiments to downregulate the expression of pro-inflammatory cytokines such as IL-6 and IL-1β (97), promote the production of anti-inflammatory cytokines such as IL-10 and TGF-β by regulating T cell differentiation. *Lactobacillus plantarum* HNU082 (Lp082) inhibits the NF-κB pathway by downregulating the mRNA expression of NF-κB2, NF-κB1, cyclooxygenase-2 (COX-2), RelA, Toll4, and iNOS, while inflammation is also regulated by NF-κB by regulating cytokine production. Additionally, UC can be alleviated by Lp082 by reducing neutrophil infiltration and myeloperoxidase (MPO) secretion (98). Probiotics can increase IL-6 secretion in a TLR2-dependent manner, induce clonal expansion of all IgA-producing B cells, promote IgA circulation, and stimulate humoral immune maturation (18, 99), and the secretion of large amounts of IgA into the intestinal lumen prevents pathogenic bacteria from reaching the intestinal epithelium and limits their colonization of the intestine (100).

**Figure 3:**
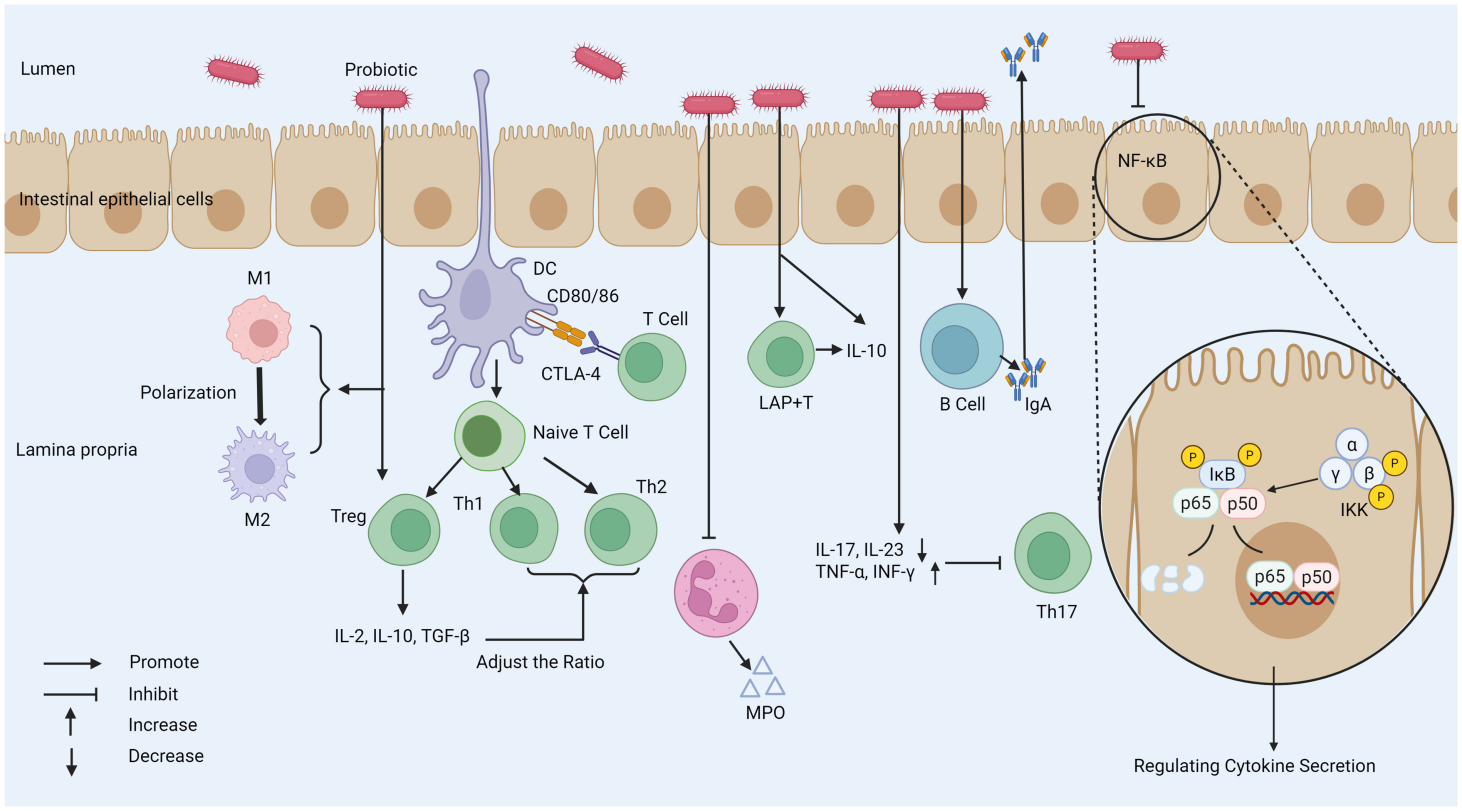
The mechanism of probiotics regulating intestinal immune response. (1) Probiotics weaken T cell response, increases the number of Tregs, and regulates the function of DC to affect the Th1/Th2 ratio. (2) Probiotics can reduce the secretion of MPO by neutrophils. (3) Probiotics promote the polarization of macrophages from the M1 phenotype to the M2 phenotype to regulate the inflammatory response. (4) Probiotics stimulate the production of IgA by B cells, and promote the IgA cycle. (5) Probiotics decrease IL-17 and IL-23 activity to inhibit Th17 activity. (6) Probiotics can inhibit the NF-κB pathway and regulate cytokine secretion. MPO, myeloperoxidase; M1, macrophage type 1; M2, macrophage type 2; CTLA-4, cytotoxic t-lymphocyte-associated protein 4; I*κ*B, inhibitor of Kappa B; IKK, I*κ*B kinase; LAP^+^T, latency-associated peptide-positive regulatory T cell; TGF-β, transforming growth factor beta; DC, dendritic cell.

Probiotics play an important role in regulating intestinal immune homeostasis through their close interaction with intestinal epithelial cells (IECs). For example, by stimulating the activation of immune cells in the intestine and regulating the production of cytokines, the immune balance in the intestinal environment can be promoted. Probiotics finely regulate immune cell function and cytokine network, which is the key molecular and cellular basis for realizing intestinal immune homeostasis reconstruction, thus exerting its anti-inflammatory and relieving IBD symptoms. This process plays a key role in inhibiting inflammatory reaction and is of great significance for relieving the symptoms of patients with IBD (Figure 3).

#### 2.3.3 Enhancement of intestinal barrier function

**Figure 4:**
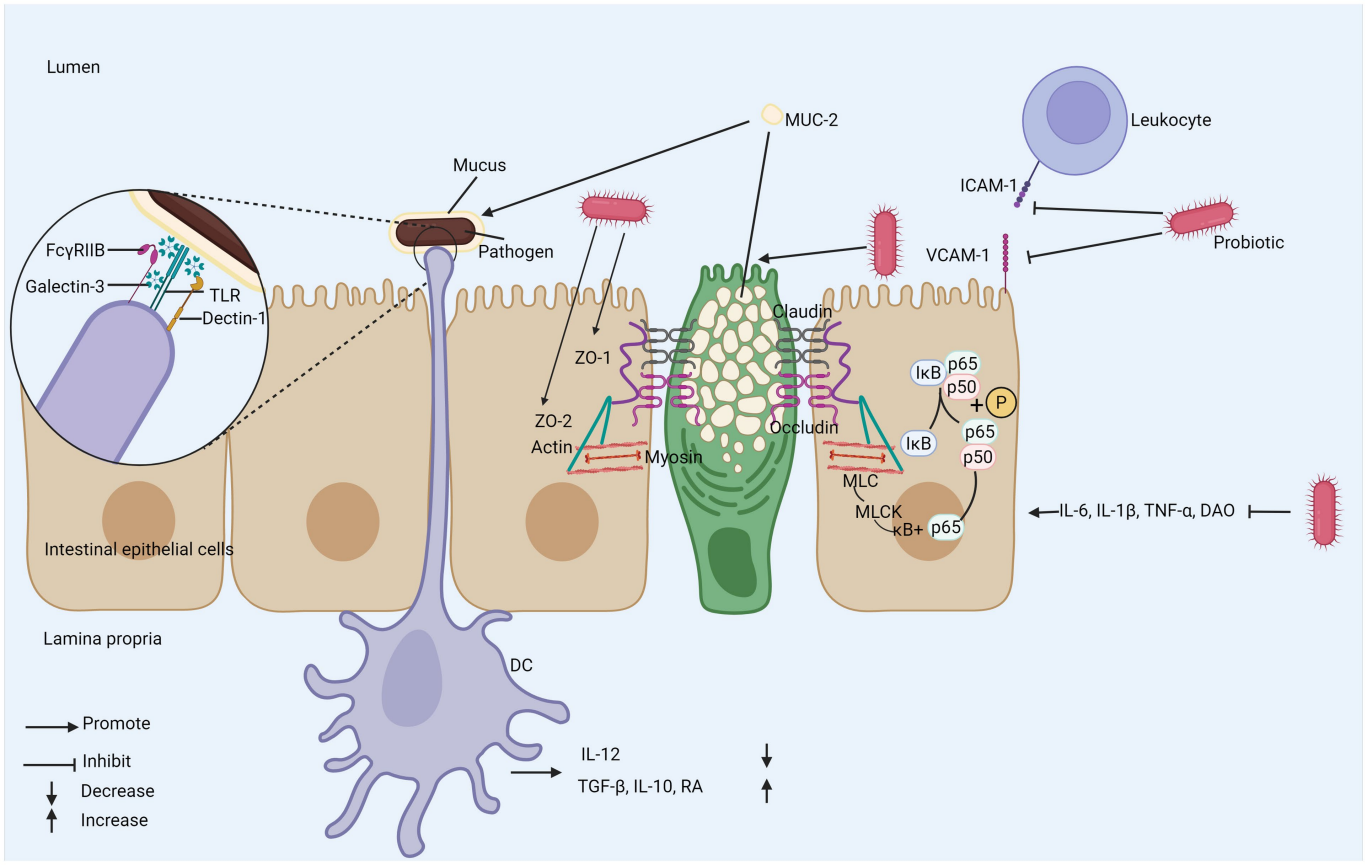
The mechanism of probiotics regulating intestinal mechanical and chemical barriers. (1) Probiotics inhibit the expression of adhesion molecules ICAM-1 and VCAM-2 to reduce damage to intestinal mucosa. (2) Probiotics promote the secretion of MUC-2 by goblet cells. (3) Probiotics promote the expression of tight junction proteins and inhibit NF-κB/MLCK to enhance intestinal barrier function. (4) DC recognizes bacteria wrapped in mucus to promote anti-inflammatory gene expression program. ZO-1; zonula occludens-1; ZO-2; zonula occludens-2; TLR; toll-like receptor; I*κ*B; inhibitor of Kappa B; MLCK; myosin light chain kinase; MLC; myosin light chain; MUC-2; mucin-2; Dectin-1; dendritic cell-associated C-type lectin-1; galectin-3; galactose-binding lectin-3; FcγRIIB; Fc gamma receptor II B; RA; retinoic acid; DAO; diamine oxidase; TGF-β; transforming growth factor beta; DC; dendritic cell.

The enhancement of intestinal barrier function is crucial to the maintenance of overall health (Figure 4). Studies in patients with IBD show that the truncated expression of O-glycans is related to the existence of the disease and the increased activity of the disease. The variation of glycan composition may destroy the mucosal layer and immune function, and eventually lead to IBD (101). Intestinal barrier consists of mechanical barrier and chemical barrier mainly composed of mucus layer, and intestinal epithelial cells and their close connection constitute intestinal mechanical barrier (102), which can effectively prevent harmful substances and pathogens from invading. Intercellular Adhesion Molecule-1 (ICAM-1) and Vascular Cell Adhesion Molecule-1 (VCAM-1) not only induce intestinal mucosal damage, but also increase intestinal mucosal permeability (103) and the mRNA expression of ICAM-1 and VCAM-1 decreases after probiotic intake.

Abnormal tight junction (TJ) proteins between intestinal epithelial cells can damage the mechanical barrier of the intestine. Studies have shown that probiotics can enhance the function of the intestinal barrier by promoting the expression of TJ proteins such as zonula occludens-1 and zonula occludens-2 (ZO-1 and ZO-2). ZO-1 and ZO-2 play important roles in the repair of the intestinal barrier (104, 105). The absence of ZO-1 and ZO-2 prevents the recruitment of occludin to tight junctions and impairs barrier function (106). *Lactobacillus paracasei* downregulates pro-inflammatory cytokines IL-6, IL-1β, TNF-α, diamine oxidase (DAO), and NF-κB p65, myosin light chain 2 (MLC2), myosin light-chain kinase (MLCK), inhibiting NF-κB/MLCK and thereby promoting barrier function (107). The chemical barrier refers to the gel-like mucin layer covering the surface of intestinal epithelial cells, with mucin-2 (MUC-2) as its main component. MUC-2 is the primary glycoprotein in intestinal mucus and is mainly secreted by goblet cells. Probiotics can stimulate the expression of MUC-2 in intestinal goblet cells (108). The mucus layer protects the host from the invasion of pathogenic microbial communities; when MUC-2 expression is reduced, the mucus loses its barrier function, which can lead to worsening of the disease (109, 110). Mucus can encapsulate bacteria and, through the action of galactose-binding lectin-3 (Galectin-3), facilitate the binding of TLRs, Fc gamma receptor II B (FcγRIIB), and Dendritic cell-associated C-type lectin-1 (Dectin-1) on DCs. This promotes the expression of anti-inflammatory gene programs, leading to decreased IL-12 expression and increased expression of TGF-β, IL-10, and RA (111). Furthermore, probiotics can increase mucus production, enhance secretion capacity, improve tissue repair ability, and inhibit apoptosis of epithelial cells, significantly improving the integrity of intestinal epithelial cells and further enhancing the function of the intestinal barrier (112, 113).

#### 2.3.4 Anti-inflammatory effects and bacterial metabolites

**Figure 5:**
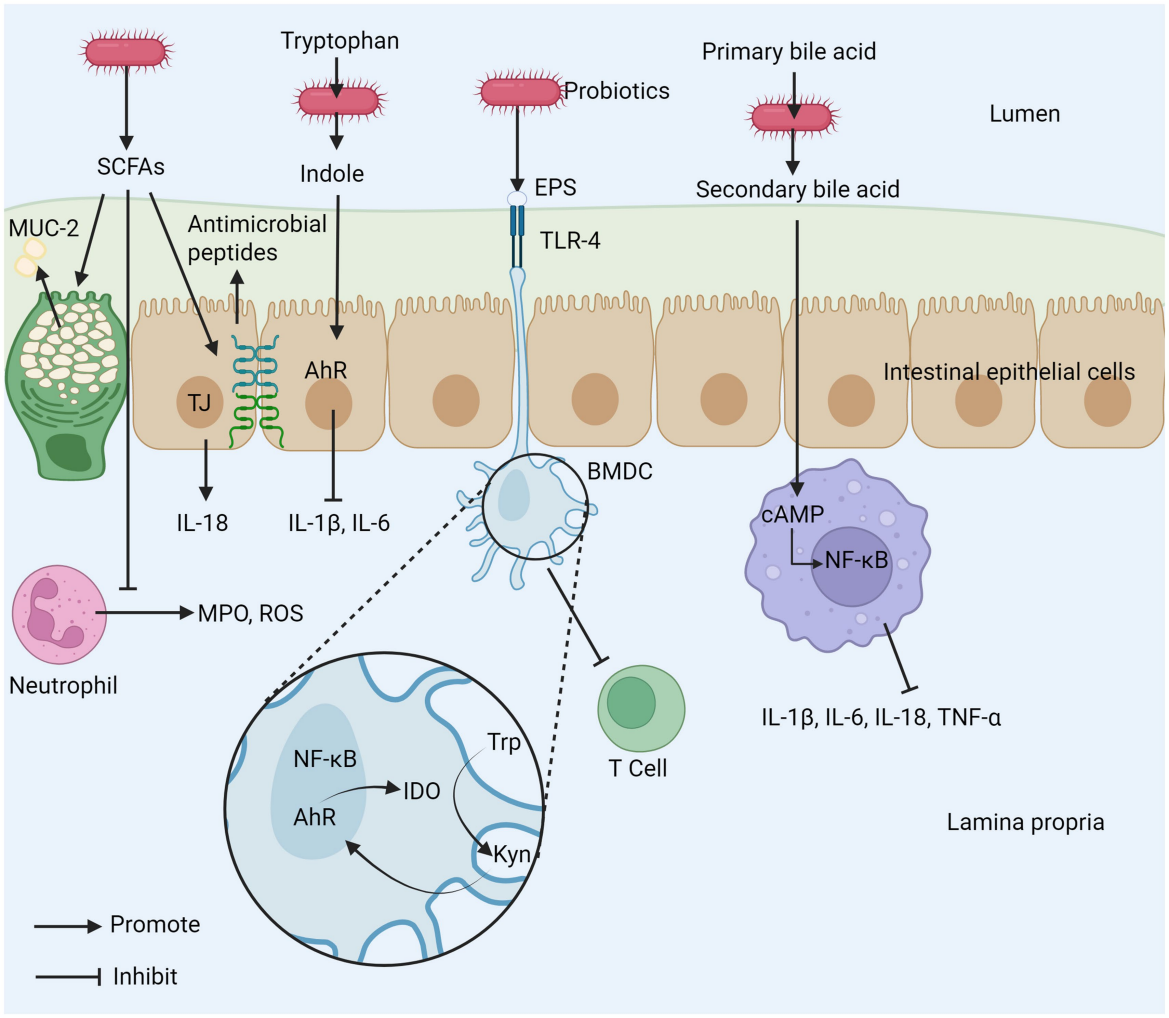
The mechanism of intestinal inflammation inhibited by probiotic metabolites. (1) SCFAs produced by probiotics can promote the secretion of MUC-2, inhibit the release of MPO and ROS from neutrophils, and promote tight junctions between cells. (2) Probiotics can metabolize tryptophan (Trp) to indole to inhibit the production of inflammatory cytokines. (3) EPS produced by probiotics can inhibit T cell activation. (4) Probiotics inhibit macrophages by metabolizing primary bile acids into secondary bile acids. SCFAs, short–chain fatty acids; EPS, exopolysaccharide; AhR, aryl hydrocarbon receptor; MPO, myeloperoxidase; ROS, reactive oxygen species; TLR-4, toll-like receptor 4; TJ, tight junction; Trp, tryptophan; Kyn, kynurenine; cAMP, cyclic adenosine monophosphate; BMDC, bone marrow-derived dendritic cell; IDO, indoleamine 2,3-dioxygenase; MUC-2, mucin-2.

The anti-inflammatory properties of probiotics are not only derived from their inherent biological activity but are also closely related to the various metabolites produced during their metabolic processes shown in Figure 5 (114). During fermentation, probiotics generate a series of short-chain fatty acids (SCFAs), such as acetate, propionate, and butyrate, which promote the integrity and permeability of the intestinal barrier in different ways (115). SCFAs can directly act on neutrophils, reducing their production of reactive oxygen species (ROS) and MPO. Butyrate increases the concentration of tight junctions by upregulating the genes encoding these proteins, such as claudin-1, ZO-1, and occludin (116). SCFAs regulate the integrity of the intestinal barrier by inducing intestinal epithelial cells to secrete interleukin 18 (IL-18) and express antimicrobial peptides; butyrate can also strengthen the mucus layer of the intestinal epithelium by increasing the expression of mucin 2 (117, 118). Additionally, SCFAs can inhibit the development of colon cancer (119). Various probiotics can metabolize to produce conjugated linoleic acid (CLA), which has been shown to have effective anti-inflammatory, immune-regulating, anti-obesity, and anti-cancer activities (120, 121). The imbalance of tryptophan (Trp) metabolism is closely related to the occurrence and development of IBD. After Trp is metabolized by bacteria into biologically active indole, it can activate the aromatic hydrocarbon receptor (AhR), thereby inhibiting IL-1β and IL-6 expression (122). A test of over 500 serum samples from IBD patients suggests that a continuous decline in Trp levels may not only promote disease progression but also further exacerbate active inflammation (123). Bone marrow-derived dendritic cells (BMDC), stimulated by the extracellular polysaccharides (EPS) of *B. subtilis*, first upregulate the immunosuppressive enzyme IDO through both classical and non-classical NF-κB dual pathways. Subsequently, signals are transmitted along the IDO-kynine-AhR axis, ultimately inhibiting T cell activation and exerting anti-inflammatory effects (124). Probiotics significantly alleviate IBD-related symptoms by improving the intestinal metabolic environment and maintaining intestinal health. Studies have shown that some probiotics can convert primary bile acids into secondary bile acids by activating the bile acid-FXR axis, and thereby exert their effects by inhibiting the NF-κB pathway in macrophages. This process can significantly reduce the expression levels of pro-inflammatory cytokines IL-1β, IL-6, IL-18 and TNF-α, thereby effectively alleviating the intestinal inflammatory response (125–127).

In summary, probiotics have many functions in IBD, such as regulating intestinal microflora, immunomodulating, enhancing intestinal barrier function and anti-inflammatory properties of its metabolites, which have laid a solid theoretical foundation for the use of probiotics as adjuvant therapy for IBD and pointed out the future research direction.

### 2.4 Clinical research and effect evaluation

#### 2.4.1 Application of probiotics in CD

CD is a chronic IBD, which is complicated and difficult to treat. The lesions may involve all parts from the mouth to the anus, and may also cause parenteral complications (128). Because of the complex pathogenesis, alternating aggravation and remission of this disease is its important feature, and the drug treatment effect is limited. In recent years, surgical intervention has played a very important role in the management and control of CD (129, 130). The imbalance of intestinal microflora in patients with CD is obvious, which will aggravate intestinal inflammation (131, 132). Probiotics may reduce this inflammation by restoring the balance of intestinal microflora (133, 134). Probiotics such as *Lactobacillus* and *Bifidobacterium* can reduce clinical activity and biomarker indicators (135).

Patients with CD often experience damage to intestinal barrier function, leading to increased intestinal permeability and exacerbating disease progression (136, 137). Studies have shown that in cells treated with probiotic mixtures, epithelial permeability measured by transepithelial electrical resistance (TEER) significantly decreases, showing that the epithelial barrier remains intact. Various probiotic mixtures protect the integrity of intestinal barrier function by boosting tight junctions and reducing the inflammatory response of intestinal cells (138, 139).

A study involving 96 patients with mildly active CD showed that mesalazine combined with a capsule containing *Bifidobacterium*, *Lactobacillus* and *Enterococcus* was more effective in treating mild active CD (140). Additionally, a review by Chen et al. of four studies involving 289 subjects reported no statistically significant difference in CD recurrence, with a *p*-value of 0.52. During the remission phase, probiotics demonstrated some efficacy (141). Bjarnason et al. studied Symprove (which contains *Lactobacillus rhamnosus* NCIMB 30173, 30174, 30175, and 30176) and found no significant changes between the placebo and probiotic groups in CD patients (142). Another experiment indicated that *S. boulardii* seems to improve CD activity index (CDAI), body mass index (BMI), serum Hb, and total cholesterol levels in CD patients during the remission phase, without safety issues (143). Bourreille et al. also studied *S. boulardii* finding that this strain is safe and well-tolerated, but it appears to have no beneficial effects on CD patients who achieved remission after steroid or salicylate treatment (144). Yılmaz et al. investigated the impact of kefir (*Lactobacillus* and *Lactobacillus kefiri*) intake on the fecal microbiota and symptoms of CD patients, discovering that regular kefir consumption may improve symptoms and quality of life in CD patients in the short term and positively affect biochemical parameters (Hemoglobin [Hgb], Erythrocyte sedimentation rate [ESR], and C-reactive protein [CPR]), significantly reducing bloating severity and increasing wellbeing index (145). In earlier experiments, Marteau et al. studied the therapeutic effects of *Lactobacillus johnsonii* LA1, concluding that this strain was well-tolerated in the study but did not significantly reduce the risk of endoscopic recurrence within 6 months after CD surgery (146). Another study on the LGG strain, conducted in pediatric subjects, showed that LGG was well tolerated and had side effects comparable to those of the placebo. When used as an adjunct to standard therapy, LGG did not prolong the time to relapse in children with CD (147).

Systematic reviews indicate that *Bifidobacterium* seems to be the only probiotic strain that is helpful, well-tolerated, and without significant side effects for CD (135). Although some studies have reported positive effects of probiotics, using probiotics for CD is still a bit controversial (148), especially since different probiotic strains exhibit varying effects, and clinical trial design and sample size are also important factors influencing research outcomes. More high-quality randomized controlled trials (RCTs) are urgently needed to further validate the efficacy and safety of probiotics in the treatment of CD (Table 2).

**Table 2:** Results of different probiotics in clinical trial for CD.

Probiotics	Placebo or other drugs	Number of people	Results	References
*Symprove* (*L. rhamnosus* NCIMB 30173, 30174, 30,175 and 30176)	Placebo	61 patients	The same analysis in the CD group showed no statistically significant changes.	(142)
*Bifidobacteria*, *Lactobacillus* and *Enterococcus*	Mesalazine	96 patients	Mesalazine combined with bifidobacteria, lactobacilli, and enterococci capsules is more effective for the treatment of mild active CD.	(140)
*S. boulardii*	/	Analysis of 92 patients among 154 cases.	*S. boulardii* can improve CDAI, BMI, serum Hb, and total cholesterol levels, safely and effectively.	(143)
Kefir (*Lactobacillus* and *L. kefiri*)	/	45 patients included with 3 voluntarily withdrew.	The experimental group showed a significant decrease in ESR and CRP. The bloating severity significantly decreased, and the wellbeing index increased.	(145)
*L. johnsonii* LA1	Placebo	98 patients	*L. johnsonii* LA1 has a poor effect on preventing the recurrence of CD.	(146)
*S. boulardii*	Placebo	165 patients	Probiotics as an adjunct treatment after standard therapy are not beneficial for maintaining remission.	(144)
LGG	Placebo	75 patients	Probiotics, as an adjunct therapy in routine treatment, are not beneficial for prolonging the time to recurrence.	(147)

#### 2.4.2 Application of probiotics in UC

A common IBD is UC, which affects the rectum and colon in different ways, mainly manifested by persistent inflammation of the colon lining, and patients usually have bloody diarrhea symptoms (149, 150). The first-line drug for mild to moderate diseases is 5-aminosalicylic acid (5-ASA). If the drug does not respond well, it may be necessary to upgrade the treatment and adopt immunosuppressive drugs, biological agents and even surgical treatment (151). Probiotics have a good prospect in the treatment of UC. Several RCTs have confirmed that specific probiotics can effectively improve the clinical symptoms and biochemical indicators of UC patients. A systematic evaluation and analysis of 33 clinical studies found that probiotics were effective in 21 studies, with strains from *Bifidobacterium* and *Lactobacillus* were regarded as the most effective choices, especially the clinical remission rate was significantly higher than that of placebo groups (135). There are great differences in the efficacy of different types of probiotics in the treatment of UC, and some studies show that the abundance of *Ligilactobacillus ruminis* in UC patients is obviously increased, which is very important for the diagnosis of the disease (152).

Most studies have shown that non-pathogenic ECN-pE are similar in efficacy and safety as mesalazine in the maintenance treatment of patients with mild to moderate UC (32). Recent studies have shown that short-term treatment with *E. coli* Nissle (ECN) is associated with reduced fecal calprotectin (FC) levels, suggesting its potential in maintaining remission in patients with UC (153).

*Lactobacillus* studies have found that Lp082 can synergistically optimize the biological, chemical, mechanical and immune barriers to improve the intestinal mucosal barrier to alleviate UC (98). In addition to strengthening the mucosal barrier, regulating the inflammatory pathway and regulating neutrophil infiltration are also potential mechanisms, Wu et al. compared the therapeutic effect of Lp082 and sulfonazioprine (SASP) on dextran sodium sulfate (DSS)-induced UC model, The results showed that Lp082 was more efficacious than SASP (98) and that UC could be alleviated by Lp082 by inhibiting NF-κB signaling molecules (154). Another study in mice showed that two strains, *Lactobacillus fermentum* GLF-217 and *Lactiplantibacillus plantarum* FLP-215, could enhance barrier function, regulate immune response, regulate intestinal microbiota composition, promote short-chain fatty acid production, and play a preventive role in DSS-induced colitis (155).

The combination of bifid triple viable capsule (*B. longum, L. acidophilus*, and *Enterococcus faecalis*) and mesalazine has been shown to improve the gut microbiota composition and reduce levels of inflammatory cytokines in IBD, with significant reductions observed in ulcerative colitis activity index (UCAI) scores and relapse rates (156). Another study divided 130 patients with UC into a treatment group (mesalazine combined with somatostatin and bifid triple viable capsule) and a control group (mesalazine combined with somatostatin). The results indicated that adjunctive therapy with bifid triple viable capsule could effectively enhance the therapeutic efficacy in UC patients, reduce plasma inflammatory factors, and modulate T cell frequencies (157). Experiments conducted by Jiang et al. also demonstrated that the combination of mesalazine and bifid triple viable capsule could enhance the treatment effect for UC, improve gut microbiota composition, attenuate immune responses, and lower levels of calprotectin (Cal) and matrix metalloproteinase-9 (MMP-9) in the intestine (158).

*S. boulardii* has attracted much attention in the fields of food and medicine due to its anti-inflammatory properties and its promoting effect on intestinal health (159). The latest research has found that heat-killed *S. boulardii* shows better efficacy in the treatment of intestinal diseases compared with live *S. boulardii* and their β-glucan components. Heat-killed *S. boulardii* can effectively repair intestinal barrier function, inhibit inflammatory responses, and regulate the balance of intestinal flora, significantly alleviating DSS-induced UC in mice (160). Furthermore, animal studies have shown that *S. boulardii* and its postbiotics can effectively alleviate the symptoms of DSS-induced colitis by regulating the host immune response and maintaining intestinal homeostasis (161).

Fecal microbiota transplantation (FMT) can effectively increase the diversity of intestinal flora in UC patients (162). Studies have shown that the combined use of FMT with probiotics rich in *Clostridium butyricum* can significantly prolong the disease remission period, and its mechanism of action may be related to the regulatory effect of butyrate. In addition, the increase in butyric acid levels in feces may serve as a potential biomarker for evaluating the therapeutic effect of FMT (163).

Probiotics are widely regarded as an intervention strategy suitable for long-term application due to their good safety and low risk of side effects (164). However, the mechanism of action and clinical efficacy of different probiotic strains in the treatment of UC still need to be verified through more high-quality clinical studies (Table 3).

**Table 3:** Results of different probiotics in clinical trial for UC.

Probiotics	Placebo or other drugs	Number of people or experimental animals	Results	References
ECN-pE	/	49 patients	After ECN-pE treatment, the FC values of patients significantly decreased, especially in UC patients, and the reduction was associated with the maintenance of clinical remission.	(153)
Lp082	DSS, SASP	Mouse	Lp082 has therapeutic effects on mouse colitis through various pathways.	(154)
*L. fermentum* GLF-217 and *L. plantarum* FLP-215	DSS	Mouse	The two strains can prevent DSS-induced colitis through various mechanisms.	(155)
Bifid triple viable capsule (*B. longum*, *L. acidophilus* and *E. faecalis*)	Mesalazine	40 patients	The combination of probiotics and mesalazine can improve the composition of the microbiota in IBD patients and reduce the levels of inflammatory cytokines.	(156)
	Mesalazine, somatostatin	130 patients	The supplementation of *Bifidobacterium* triple live bacteria capsules can enhance the efficacy against UC.	(157)
	Mesalazine	180 patients	Mesalazine combined with a *Bifidobacterium* triple therapy can enhance the efficacy of UC.	(158)
*S. boulardii*	DSS	Mouse	Heat-inactivated *S. boulardii* alleviates DSS-induced UC in mice.	(160, 161)

#### 2.4.3 Results and analysis of existing clinical trials

At present, the results of clinical trials are encouraging. Probiotics have a good potential in the treatment of IBD. Systematic evaluation shows that probiotics are effective in relieving UC. Some studies even suggest that probiotic supplements can replace conventional drug therapies (165–167). However, the research on CD is not only relatively few, but also controversial. Some studies have not shown significant clinical improvement (135, 148, 165, 168), but probiotics still have therapeutic potential in this field.

Studies have shown that the efficacy of probiotics is usually strain-specific, and the therapeutic effect may be significantly different when different strains treat different diseases (169, 170). In the future, more attention should be paid to specific strains, the optimal dosage and the combination with other therapies to determine more effective treatment strategies.

At present, the number of related clinical trials is limited and the sample size of many existing studies is small, which limits the universality of research results. In the future, we need to be stricter in clinical trial design to comprehensively assess the actual efficacy and safety of probiotics, providing patients with more reliable treatment options.

### 2.5 Future prospects of probiotic treatment for IBD

After in-depth exploration of the role of probiotics in the treatment of IBD, the future of probiotics looks bright and exciting. Probiotics can effectively improve gut microbiota dysbiosis and modulate immune responses to alleviate inflammation, offering IBD patients a novel and potentially effective treatment strategy (171).

The key directions of future research are personalized probiotic treatment regimens, the development of new probiotics, and its combination with other therapeutic approaches, which require extensive attention and discussion from the scientific community and clinical medicine (172, 173).

#### 2.5.1 Personalized probiotic treatment

The increasing importance of personalized probiotic therapy for IBD, which may be more effective due to significant differences in gut microbiota and immune responses in different patients (174).

Selecting the right probiotic strain based on the specific characteristics of the patient’s gut microbiota can significantly improve the efficacy of treatment, and one study involving 24 adults showed that receiving *B. longum* BB536 was demonstrated (175). Dietary habits and the types and quantities of gut microbiota vary greatly among populations in different regions, which may lead to different outcomes from the same probiotic treatment. One study involving 66 patients with an average age of 46.2 years, of whom 53% were female and 89.4% were white, observed a trend between probiotic use and brain fog (*p* = 0.080), but it did not reach statistical significance. However, in white patients, the association between probiotic use and brain fog reached statistical significance (*p* = 0.044), and the study found a significant statistical association between probiotic use and brain fog symptoms in male patients (*p* = 0.004), with the duration of probiotic use also related to the occurrence of brain fog (*p* = 0.038). These results suggest that probiotic use may be significantly associated with brain fog symptoms in specific populations, such as white and male patients, and that the duration of use may also influence this relationship (176). Multiple studies have also shown that the efficacy of probiotic treatment varies among different age groups. An analysis of nine trials showed no significant effect of probiotics on CD (*p* = 0.07), but three trials involving children with IBD demonstrated significant advantages (*p* < 0.01) (177).

Research indicates that IBD severity is closely linked to specific bacterial biomarkers in fecal microbiota. Quantitative polymerase chain reaction (qPCR) is an effective method for detecting these biomarkers, aiding in monitoring IBD progression (178). Studies show that using probiotics derived from an individual’s own gut microbiota, rather than commercial options, can better treat DSS-induced colitis by reducing disease susceptibility and enhancing immune response (179). Future integration of genomics and metabolomics is expected to advance personalized probiotic therapies, improving treatment outcomes and patient quality of life.

#### 2.5.2 Research directions for new probiotics

With the continuous development of probiotics, new probiotics have opened up a new direction for the treatment of IBD and shown us unprecedented potential. In the future, with the continuous progress of genetic engineering technology, the emergence of engineered probiotics will also provide new hope for the treatment of IBD (180, 181).

Genetically engineered probiotics are a leading field in the treatment of IBD, on the one hand, they can regulate intestinal dysbiosis, and on the other hand, they can also release therapeutic active molecules directly into the intestine, which can effectively avoid the side effects caused by systemic administration (182). Due to the high genetic stability and non-transferability of small recessive plasmid mutant 1 (pMUT1) and mutant 2 (pMUT2), *E. coli* is commonly used as genetically engineered bacteria, particularly ECN (183). For example, genetically engineered ECN-pE can eliminate harmful reactive oxygen species (ROS) in the gut, show significant anti-inflammatory effects in mouse IBD models, and successfully repair the intestinal epithelial barrier, significantly increasing the abundance of probiotics (184). Liu and colleagues engineered *Bifidobacterium* expressing the PEP-1-hMnSOD fusion protein, which can successfully express rhMnSOD in the colon, to treat DSS-induced UC in mice.

Results indicate that engineered *Bifidobacterium* effectively reduces DSS-induced UC, as evidenced by the levels of inflammatory cytokines TNF-α, IL-1β, IL-6, and IL-8 in colonic tissue and histological damage (185). A strain of *Lactobacillus paracasei* F19 (pNAPE-LP) expressing N-acyl phosphatidylethanolamine-specific phospholipase D (NAPE-PLD) was developed by Esposito G et al. And under the promotion of ultra-low palmitate supply, the strain can produce palmitoyl ethanolamine (PEA), and when pNAPE-LP is co-administered with palmitate, PEA will be released over time, thus significantly improving the clinical and histological injury score in UC mouse model, and significantly reducing neutrophil infiltration, reducing the expression and release of pro-inflammatory cytokines and oxidative stress markers, and enhancing the integrity of epithelial barrier. PNAPE-LP is a new therapy to control intestinal inflammation in IBD (186). These genetically engineered probiotics can not only release therapeutic molecules in the intestine to enhance the anti-inflammatory effect, but also provide new ideas for future treatment strategies.

Recent studies have shown that the application of nanocoating of probiotics has attracted widespread attention. Due to the harsh intestinal environment, the feasibility and effectiveness of probiotic therapy may be affected, and modifying probiotics with nanocoating can enhance their resistance to the gastrointestinal environment (187, 188). A study encapsulated probiotic *Bacillus coagulans* spores with rosmarinic acid (RA) and silk fibroin protein (SF), among them, RA can clear ROS to alleviate oxidative damage and inhibit inflammatory response, while SF can assist in the colonization of probiotics. This probiotic alleviates a series of inflammatory symptoms and restores the balance of gut microbiota (189). In summary, such research may open up new avenues for probiotic treatment of IBD in the future.

#### 2.5.3 Combined application of probiotics with other treatment methods

The use of probiotics alongside other treatments shows a lot of promise in providing more comprehensive and personalized treatment options for IBD patients (190).

Currently, traditional treatment methods such as immunosuppressants and biologics, while effective, often come with significant side effects (191), causing inconvenience and distress for patients. Therefore, incorporating probiotics into adjunctive treatment plans could help lower the doses of these medications and effectively reduce the incidence of their adverse reactions. Combining probiotics with other treatment methods can really boost treatment results and play an important role in improving patients’ quality of life (192). Additionally, synbiotics are emerging as a promising new approach with potential in treating IBD. Synbiotics are a combination of probiotics and prebiotics, with prebiotics being indigestible food components that selectively stimulate the growth of beneficial bacteria or promote the activity of a limited number of health-promoting bacteria (30, 193). For example, research by Xue et al. found that Lp90 and soluble dietary fiber (SDF) obtained from mushroom byproducts, as synbiotics, can upregulate butyrate production and increase gut microbiota diversity to alleviate colitis (194). Other studies combined *Lactobacillus plantarum* SC-5 (SC-5) and tyrosol (TY), with this synbiotic alleviating and improving colitis in a gut microbiota-dependent manner (195). Relevant systematic reviews indicate that synbiotics significantly improved patients’ colonoscopy and histological scores, clinical activity index, serum CPR levels, gut microbiota index, and levels of messenger RNA, TNF-α, IL-1α, IL-10, and MPO (196). In summary, synbiotics can help in treating IBD in different ways (197, 198). Future research should investigate how probiotics can be combined with other treatments to improve IBD management and enhance patient health outcomes.

## 3 Conclusion

The medical community is increasingly recognizing and engaging in discourse regarding the significant role and potential application of probiotics in the management of IBD. Recent studies have demonstrated that probiotics can modulate intestinal microbiota, enhance the integrity of the intestinal barrier, regulate immune responses, and exert anti-inflammatory effects, thereby ameliorating symptoms and enhancing the quality of life for patients with IBD.

Although the application prospects of probiotics in IBD treatment are broad, we still need to pay close attention to the differences in research results. On one hand, some studies indicate that specific probiotic formulations can significantly improve patients’ clinical symptoms and quality of life; on the other hand, other studies have failed to find significant therapeutic effects. This variation might be due to several factors, including differences in study design, individual participant differences, types of probiotics, and their dosages. Therefore, future research should focus more on standardization and personalization in order to more accurately assess the actual effects and application value of probiotics in IBD treatment.

The research direction of probiotics in the treatment of IBD is expected to develop in the direction of personalized treatment, the development of new probiotics and the combined application of other treatment methods in the future, to improve the treatment effect, personalized treatment may have to be adjusted according to the characteristics of the patient’s intestinal flora to determine the best combination of probiotics, and the research of genetically engineered probiotics and synbiotics also brings new ideas to the management of IBD, and the application prospects and research value of probiotics in the adjuvant treatment of IBD are broad, and it is estimated that they will play a more important role in the treatment of IBD. This provides patients with better treatment options and improves their quality of life.

## Glossary

AhR: Aryl hydrocarbon receptor
BMDC: Bone marrow-derived dendritic cell
BMI: Body mass index
CD: Crohn’s disease
CDAI: Crohn’s disease activity index
CPR: C-reactive protein
CTLA-4: Cytotoxic t-lymphocyte-associated protein 4
DAO: Diamine oxidase
Dectin-1: Dendritic cell-associated C-type lectin-1
DFC: Dietary fiber complexes
DSS: Dextran sulfate sodium
ECN: *E. coli* Nissle
EGC: Enteric glial cells
EPS: Exopolysaccharide
ESR: Erythrocyte sedimentation rate
FC: Fecal calprotectin
FcγRIIB: Fc gamma receptor II B
FimM: Fimbrial m protein
FMT: Fecal microbiota transplantation
Galectin-3: Galactose-binding lectin-3
GDNF: Glialcell-derived neurotrophic factor
Hgb: Hemoglobin
IBD: Inflammatory bowel disease
IBS-C: Irritable bowel syndrome with constipation
ICAM-1: Intercellular cell adhesion molecule 1
IDO: Indoleamine 2,3-dioxygenase
IgA: Immunoglobulin A
IκB: Inhibitor of kappa B
IL-1β: Interleukin 1β
IL-10: Interleukin 10
IL-17: Interleukin 17
IL-18: Interleukin 18
IL-23: Interleukin 23
Kyn: Kynurenine
LAP+T: Latency-associated peptide-positive regulatory T cell
Lp082: *Lactobacillus plantarum* 082
MAPK: Mitogen-activated protein kinase
MHC-II: Major histocompatibility complex class II
MLC: Myosin light chain
MLCK: Myosin light chain kinase
MPO: Myeloperoxidase
MUC-2: Mucin-2
NCFM: Matrix metallopeptidase 9
NF-κB: Nuclear factor kappa B
PEA: Palmitoyl ethanol amide
RCT: Randomized controlled trial
ROS: Reactive oxygen species
SASP: Sulfasalazine
SCFAs: Short-chain fatty acids
SF: Silk fibroin protein
TGF-β: Transforming growth factor-β
TLR: Toll-like receptor
TLR-2: Toll-like receptor 2
Th17: T helper cell 17
TJ: Tight junction
TNF-α: Tumor necrosis factor-α
Trp: Tryptophan
UC: Ulcerative colitis
VCAM-1: Vascular cell adhesion molecule 1
ZO-1: Zonula occludens-1
ZO-2: Zonula occludens-2
